# Colorectal carcinoma associated with schistosomiasis: a possible causal relationship

**DOI:** 10.1186/1477-7819-8-68

**Published:** 2010-08-13

**Authors:** Omer E H Salim, Hytham K S Hamid, Salwa O Mekki, Suleiman H Suleiman, Shakir Z Ibrahim

**Affiliations:** 1 Department of Surgery, Faculty of Medicine, University of Khartoum, Khartoum, Sudan; 2 Department of Surgery, Soba University Hospital (SUH), Khartoum, Sudan; 3 Department of Histopathology, Soba University Hospital (SUH), Khartoum, Sudan

## Abstract

The association between schistosomiasis and colorectal malignancy has long been suggested in the literature, but it is not uniformly accepted. In the Far East, considerable evidence supports an etiological link between *Schistosoma japonicum *and colorectal cancer. However, the available data regarding the role of *Schistosoma mansoni *in colorectal carcinogenesis are conflicting and most often do not show causality. We report on a patient with sigmoid colonic cancer coexisting with schistosomiasis, and we provide a comprehensive review of the literature regarding the epidemiology and pathobiology of this association.

## Background

Schistosomiasis is a fairly prevalent communicable disease in tropics and subtropics caused by a trematode of the genus schistosoma. It affects more than 200 million people worldwide, with over 700 million living under conditions favouring transmission [1]. Human schistosomiasis is generally caused by three major species: *Schistosoma mansoni *(*S. mansoni*) endemic in Africa, the Middle East, and South America, *Schistosoma japonicum *(*S. japonicum*) common in Southeast Asia, and *Schistosoma haematobium *(*S. haematobium*) prevails in Africa and the Middle East [1].

In endemic areas, schistosomal infestation has been implicated in the aetiology of several human malignancies including bladder, liver, and colorectal cancer (CRC) [2]. However, while sufficient evidence supports a causal relationship between *S. hematobium *infection and bladder cancer, the association between schistosomal infestation and CRC has apparently low status within the canons of medicine and reports from the publishing world [3]. Furthermore, most of the published data refer to *S. japonicum *species, whilst the evidence linking *S. mansoni *to CRC occurrence is meagre.

We herein present a case of sigmoid colonic adenocarcinoma associated with deposited *S. mansoni *ova, and we discuss the probable etiological role of chronic schistosomal infestation in colorectal cancer.

## Case Report

A 35-year-old man presented with four-year history of left lower crampy abdominal pain, constipation, and occasional bleeding per rectum. He ever went to clinic for help with symptomatic improvement after empirical treatment, whereas no definite diagnosis was made. In the last two months, his abdominal pain got worse and the rectal bleeding became more frequent and almost constant. He also had anorexia and moderate weight loss. His past history was notable for appendecectomy and inguinal hernia repair. The patient came from Al-Gezira province, an area of high endemicity for schistosomiasis. He was previously diagnosed with schistosomal infection at age of 20 through stool examination. He received antischistosomal treatment, however, no further tests were done to confirm cure at that time.

Physical examination was insignificant apart from surgical scars on the abdomen. Laboratory studies disclosed the following values; white blood cell count 5,200/mm^3 ^with neutrocyte/lymphocyte 76/19, without eosinophilia; hemoglobin 13.1 g/dl; platelet 292,000/mm^3^; albumin 3.7 g/dl; total bilirubin 2.1 mg/dl; ALT 32 U/L; AST 39 U/L. Renal function and electrolytes were within normal limits. Three consecutive stool tests were negative for *S. mansoni *ova. Preoperative carcinoembryonic antigen (CEA) level was 4.27 ng/mL (normal range < 5 ng/mL). Abdominal sonography did not show significant periportal fibrosis or splenomegaly. An abdominopelvic computed tomographic scan showed thickening of the sigmoid colon wall, with no evidence of lymphadenopathy or liver metastases. Endoscopic examination of the colorectum demonstrated an ulcerating lesion in the sigmoid colon. A biopsy was performed and the pathological report showed mucinous adenocarcinoma.

**Figure 1:**
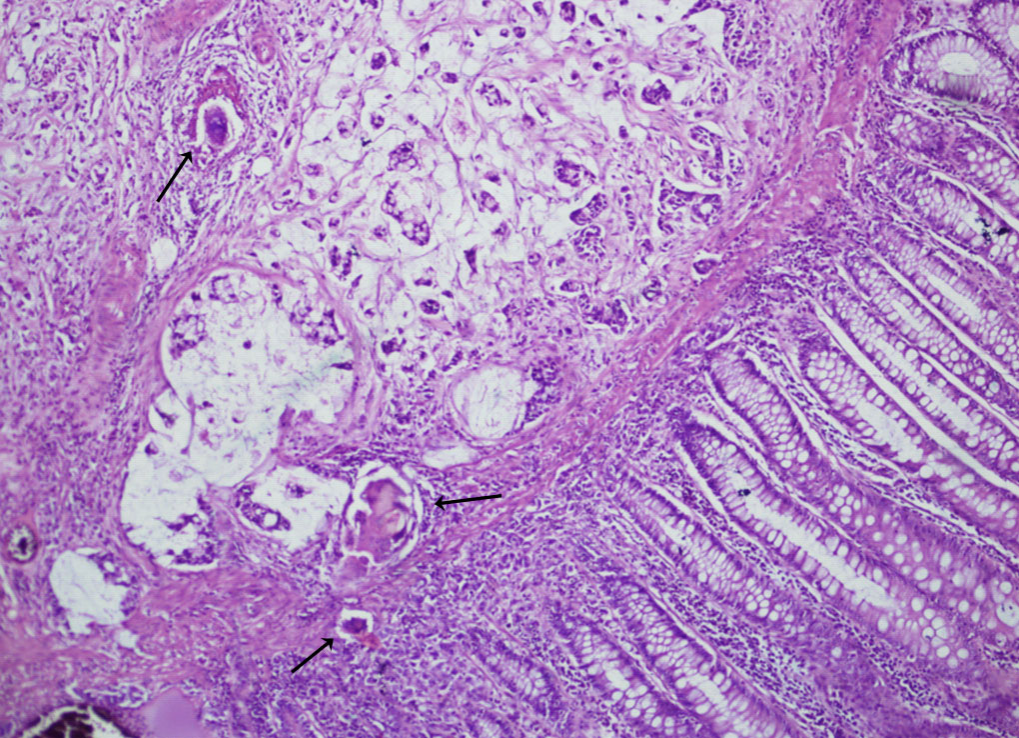
**Photomicrograph showing mucinous adenocarcinoma deep to normal colonic epithelium, and calcified *S. mansoni *ova (black arrows) inside and outside the tumour**. H&E × 40.

**Figure 2:**
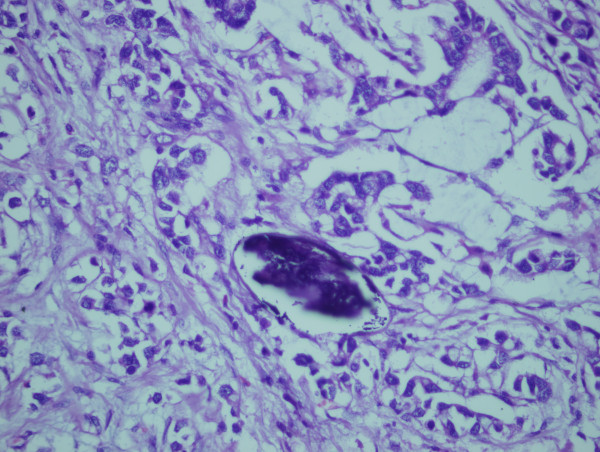
**Photomicrograph showing typical *S. mansoni *egg with a characteristic lateral spine in a background of mucinous adenocarcinoma**. H&E × 40.

**Figure 3:**
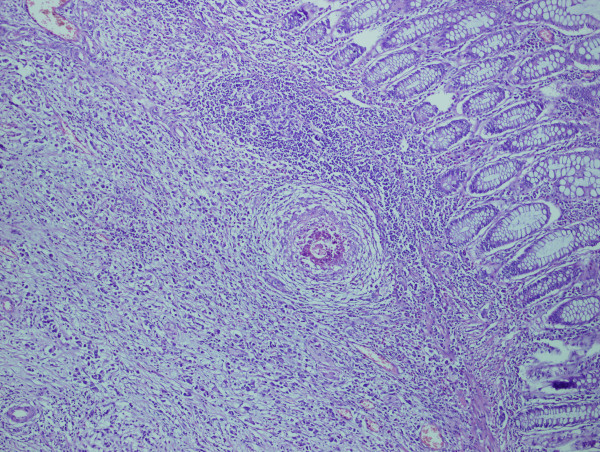
**Photomicrograph revealing calcified *S. mansoni *ova and granuloma formation in the submucosa of the sigmoid colon**. H&E × 40.

The patient underwent anterior resection for sigmoid colon cancer. Gross examination of the resected specimen showed ulcerated tumour measuring 5 × 4 cm. Succedent pathological analysis of the specimen revealed mucinous adenocarcinoma infiltrating the muscularis propria without evident serosal involvement. Deposited ova of *S. mansoni *were found in the cancerous tumour (Figure 1, 2) and in the colonic submucosa (Figure 3). The eggs were more often seen in the tumor than in the normal tissue. The postoperative pathological staging was consistent with pT2, N0, M0.

The patient had an uneventful postoperative course and was discharged on the 11^th ^day. He had no clinical or endoscopic evidence of recurrence on the 6^th ^follow-up visit two years later.

## Discussion

The schistosome parasite is a digenetic blood-dwelling fluke, of the flatworm variety, which has a definitive mammalian host and an intermediate snail host. The adult worms of the main intestinal species, *S. mansoni *and *S. japonicum*, are found in pairs in the mesenteric vessels, where they lay their eggs. The eggs penetrate the intestinal wall and are shed in the stool of human or other vertebrate host. Upon contact with fresh water, the excreted eggs hatch and release miracidia which, after infecting the appropriate snail host, multiply asexually into cercarial larvae. Following penetration of the human skin, the cercarial larvae transform into schistosomulae and undergo maturation in the portal vein. The mature male and female worms typically mate and inhabit the mesenteric venules in various locations, which at times seem to be specific for each species. Eventually, gravid female worms release eggs, which traverse the intestinal wall to reach faeces and renew the cycle. Many ova, however, are retained in the gut wall particularly the rectum, or flow backward and cause egg embolism in the liver or other organs. In the intestine, the sequestered eggs in the mucosa and submucosa incite a severe inflammatory reaction with cellular infiltration and consequent granuloma formation. This in turn leads to mucosal ulceration, microabscess formation, polyposis, and neoplastic transformation [4].

### Epidemiological evidence of association

The epidemiologic parallel between schistosomiasis japonica endemicity and the distribution of large bowel cancer has been noted in the eastern provinces of China in the 1970 s [5]. Subsequently, ecological studies in the same endemic areas showed a strong geographical correlation between the prevalence of schistosomiasis japonica and CRC incidence and mortality [6]. Likewise, significant association was observed between the mortality from CRC and from schistosomiasis japonica in rural China, even after adjustment for dietary factors [7,8]. The authors attributed the continuing high incidence of colorectal cancer in endemic regions to persistent large populations of chronically infected individuals. This conclusion was further bolstered by a retrospective cohort study conducted in an endemic area in Japan, where the standardized mortality ratio for colonic cancer was significantly high in females who lived in the area for 50 years or more [9].

More importantly, a case-control study carried out in the endemic area of Jiangsu Province, China, evidenced that the risk of rectal cancer was increased among subjects with a previous diagnosis of *S. japonicum *infection with odds ratios of 4.5 and 8.3 (depending on the type of controls used), but the risk of colon cancer was not significantly increased in the same patients group [6]. In a similar investigation in the same endemic area, Guo et al. confirmed strong associations between colon cancer and early and late-stage *S. japonicum *infection, regardless of the type of control used for comparison. When the results were adjusted to smoking and family history of colon cancer, statistically significant associations were still noted. In addition, the estimated relative risk increased with the duration of exposure to *S. japonicum *infection [10]. Of interest also is a recent matched case-control study which reported that patients with chronic schistosomiasis japonica have more than three times risk to develop colon cancer than those with no previous exposure to schistosomal infection. Moreover, the authors attributed 24% of colon cancer cases to long-standing schistosomal infestation [11].

The epidemiological evidence associating *S. mansoni *infection with CRC is lacking, of poor quality, or conflicting. Supporting the absence of such a causal association, Parkin pointed out that although there is a great disparity in the geographical distribution of *S. mansoni*, CRC occurs in the African continent with clear uniformity [12]. In a recent hospital-based study in Uganda and Zimbabwe, Waku and colleagues compared 950 cases of infective gastrointestinal disease, particularly schistosomiasis and amebiasis, with 249 patient controls admitted for various diseases other than GI disease. The cases were thoroughly investigated and further stratified into three groups on the basis of the stage of the disease; cured, acute, and chronic patients group. Colorectal cancer was found in 34 patients; nearly all of them had chronic schistosomiasis or amebiasis, whereas no CRC was detected in the other patients or control groups. It was concluded that large bowel cancer is strongly associated with chronic infectious gastrointestinal diseases [13]. This study, though, was limited by the inability to adjust for potential confounders such as age and gender. Furthermore, the issue of correspondence between the population giving rise to the cases and that sampled for the controls was not addressed. To date, there have been no epidemiological studies conducted at the population level to verify the link between *S. mansoni *infestation and large bowel cancer.

### Clinicopathological data

The consensus of available pathological data strongly implicates an association between *S. japonicum *infestation and induction of CRC. In a review of the literature between 1898 and 1974, 276 cases of schistosomiasis japonica associated with cancer of the large intestine were analysed. The results showed significant differences between carcinoma with schistosomiasis and ordinary carcinoma in symptoms, age range, sex ratio, and histopathologic findings, indicating that schistosomiasis may induce carcinoma [14]. Chen et al. reported similar findings in their study of 90 cases of simultaneous CRC and schistosomiasis, and proposed that *S. japonicum *colitis, in its late phases, is a premalignant condition not infrequently leading to cancer [15]. Supporting their previous results and giving better insight into the pathogenesis of schistosomal colorectal carcinoma, the same authors examined the mucosal changes in the immediate vicinity of the tumours of patients with schistosomiasis, and referred to the close similarity between certain schistosome-induced lesions and those associated with long-standing ulcerative colitis. Pointing to mimicry of cancer evolution in these two clinical entities, they described presence of pseudopolyps, multiple ulcers, and hyperplastic ectopic submucosal glands, with evidence of oviposition and precancerous and cancerous transformation in these lesions [16]. In addition, it was demonstrated that the closer to the tumour the area is the more ova tend to be detected [17]. In a following study, Chen et al. observed variable degree of colonic epithelial dysplasia in 60% of cases with *S. japonicum *colitis and regarded these changes as the transition on the way towards cancer development in schistosomal colonic disease [18]. A similar conclusion was drawn by Yu et al. from their studies on different types of schistosomal egg polyps [19].

Several case reports and descriptive studies from Africa and the Middle East have raised the possibility of an association between *S. mansoni *infestation and colorectal carcinoma [20-23]. Nonetheless, the pathological evidence supporting this claim is rather weak. In 1956, Dimmette et al. failed to demonstrate any specific pathological changes in patients with simultaneous CRC and *S. mansoni *infestation, and considered the two conditions unrelated [24]. Contrasting to these results, a recent study by Madbouly et al. has shown that S. mansoni-associated colorectal cancer has distinctive pathological features often similar to those of colitis-induced carcinoma. These include high percentage of multicentric tumours and mucinous adenocarcinoma, and the tendency of the tumour to present at an advanced stage with high risk of malignant lymph node invasion [25]. Although direct causal inference is limited, this study indicates that *S. mansoni *infestation may exercise some influence on the prognosis of patients with CRC. Other studies have examined the pathological changes in endoscopic biopsies and cadaveric specimens from the colon of patients with *S. mansoni *colitis [26,27]. The gross pathological lesions were akin to those observed in patients with *S. japonicum *colitis. However, histological analysis of the specimens showed no evidence of atypism or carcinomatous changes. This discrepancy in pathologic findings may be explained by the larger number of eggs deposited by *S. japonicum *than *S. mansoni *worms, thus causing more pathological problems [28].

In general, there are some unique characteristics of schistosomal colorectal cancer that seem to already be emerging from the existing literature, regardless of the associated schistosomal variant. Bearing in mind the early environmental exposure to schistosomal infection in childhood, schistosomal colorectal cancer was notably shown to occur in younger age group with a maximum age incidence 6 to 16 years earlier than ordinary colorectal cancer [14-16,25]. Furthermore, the gender ratio of male to female in schistosomal colorectal cancer is consistently higher than in non-schistosomal cancer [14,16,25]. This can be attributed to the fact that men are more prone to schistosomal infection through contact with cercariae-infested waters during agricultural activities. Pathologically, schistosomal colorectal carcinomas appear to have a strong predilection for the rectum [17,25], and they quite frequently have a mucinous histology [25,29,30]. In our index case, the young age of the patient and the presence of higher density of *S. mansoni *ova within the cancerous tumour rather than in the normal tissue point to a strong association between *S. mansoni *infection and colorectal cancer.

### Pathogenesis and Molecular events

The exact etiopathogenesis of schistosomal colorectal cancer is enigmatic. Several explanations have been advanced for the possible role of schistosomiasis in colorectal tumorigenesis: the presence of endogenously produced carcinogens [31], chronic immunomodulation resulting in impairment of immunological surveillance [32], symbiotic action of other infective agents [14], and the presence of schistosomal toxins [33]. While these factors may interact to induce carcinogenesis, chronic inflammation appears to play a central role. In support of this view are data showing that CRC tends to occur mainly in patients who had history of schistosomiasis for 10 years or more and in whom the large bowel is wholly involved [14,16]. Moreover, there is significantly higher rate of synchronous tumours in patients with schistosomal colorectal cancer than in patients with spontaneous colorectal cancer [16,25]. This can be ascribed to the field effect caused by chronic schistosomal inflammation throughout the colon, a phenomenon analogous to that described in the context of colitis-associated cancer.

It has been suggested that chronic inflammatory reaction provoked by schistosome antigens provides the proliferative stimulus necessary to promote cancer growth from potentially malignant foci produced by other carcinogens [16]. However, whereas increased epithelial cell proliferation likely contributes to carcinogenesis, it is insufficient to cause cancer. Rather, inflammatory cells generate potentially genotoxic mediators during the course of schistosomal infection such as reactive oxygen and nitrogen species and proinflammatory cytokines, which cause genomic instability and dysregulation of oncogenes and oncosuppresor genes [34,35]. The accumulation of these molecular disturbances, in turn, drives the progression toward dysplasia and carcinoma. Another factor that may play a major role in colorectal carcinogenesis of schistosomiasis patients is the presence of concomitant enterobacterial infections. In both clinical and experimental studies, various strains of enterobacteriaceae have been described in association with schistosome infection which confers a survival advantage to bacteria by inducing immunosuppression [36,37]. Some of these organisms are thought to promote colorectal carcinogenesis through multiple pathways such as production of reactive oxygen intermediates, dysregulation in the T cell response, and alterations in host epithelial carbohydrate expression [38].

A further explanation for the carcinogenic process of schistosomal CRC is a possible direct mutagenic effect of the schistosome soluble antigens. Evidence against this hypothesis has come from a study by Ishii et al. [39], who evaluated the mutagenicity of *S. japonicum *extracts using the Ames Salmonella/E. coli test in the presence and absence of rat liver S9 mixture. They did not identify any mutagenic activity for the soluble extracts of both eggs and adult worms. Nevertheless, a weak but significant tumour-promoting activity was noted for the *S. japonicum *soluble egg antigen when tested using cultured viral genome-carrying human lymphoblastoid cells [39]. Osada et al. tested the adult worm and egg extracts of *S. mansoni *using more reliable genetic toxicology assays, the Salmonella Umu test and the hypoxanthine guanine phosphoribosyltransferase (HGPRT) gene mutation assay. They could not demonstrate any mutagenic potential in either parasite extracts of *S. mansoni *before and after addition of S9 mixture [40].

Recent studies have thrown some light on the molecular events associated with schistosomal colorectal cancer, taking the latter as a separate clinical entity. Zhang et al. investigated the mutation pattern in the p53 gene in S. japonicum-associated rectal carcinomas. They observed a higher proportion of base-pair substitutions at CpG dinucleotides and arginine missense mutations among schistosomal rectal cancer patients than in patients with ordinary CRC, albeit the differences were of marginal significance. Their results also indicated that the majority of mutations in p53 gene were in exon 7 in schistosomal group compared to exon 5 in non-schistosomal group [41]. Barrowing from the ulcerative colitis example, nitric oxide, an endogenously produced genotoxic agent, is capable of inducing similar transition mutations and activation of P53 gene in the inflamed colonic mucosa [42]. Conceivably therefore, it seems plausible that chronic colonic inflammation induced by schistosomal infection may follow a similar pathway.

For *S. mansoni-*associated colorectal carcinomas, it was demonstrated that parasitism is strongly associated with microsatellite instability, which is a sign of defective DNA repair [30]. This genomic instability results in DNA replication errors that preferentially affect target genes such as transforming growth factor (*TGH*)*βRII *and insulin-like growth factor (IGF)2R, and render them incapable of normal colonocytes homeostasis resulting in malignant growth [43]. In another aspect, Madbouly et al. evaluated the expression of p53 in patients with *S. mansoni*-related colorectal cancer, and found that mutant p53 overexpression was significantly more frequent in schistosomal than in non-schistosomal colorectal cancer. Moreover, p53 overexpression in schistosomal CRC correlated well with mucinous carcinoma, nodal metastasis, and tumour multicentricity [25]. Zalata and his associates developed a more comprehensive study of the expression pattern of p53, Bcl-2, and C-Myc in seventy five CRC cases, 24 of these had pathological evidence of *S. mansoni *infection. Although they did not find a significant association between parasitism and p53 and C-Myc expression, their results showed that S. mansoni-associated colorectal tumours characterize by Bcl-2 overexpression and less apoptotic activity than ordinary colorectal tumours [44]. This supports the contention that evasion of apoptosis through change in the expression of Bcl-2 may be an alternative molecular pathway through which genotoxic agents can induce carcinogenesis in intestinal schistosomiasis.

It is clearly evident that multiple genetic changes occur during the development of schistosomal colorectal carcinoma, and while some of these changes may play an integral role in tumour progression, others appear to have a significant impact on the prognosis. The molecular profile of schistosomal colorectal carcinomas is in part different from what has been demonstrated in colitis-induced carcinomas, implying that factors other than inflammation are involved in the carcinogenic process.

## Conclusion

Even though the evidence for a definite aetiological association between schistosomal infestation and large bowel cancer is currently inconclusive, the compelling epidemiological data and the unique clinicopathological features of schistosomal colorectal cancer described hitherto, entail a possible role for chronic schistosomiasis in promoting carcinogenesis of colorectal neoplasms. The pathogenetic mechanisms underlying schistosomal colorectal cancer are far from clear, nevertheless the pathobiological similarities to colitis-induced carcinomas points to inflammation as a key factor in the carcinogenic process. We believe that the available data only allow one to consider S*. japonicum *as a probable carcinogen in humans, leading to colorectal cancer, whereas further epidemiological and experimental studies are warranted to investigate the cause and effect relationship between *S. mansoni *and colorectal malignancy.

## Consent

Written informed consent was obtained from the patient for publication of this case report and any accompanying images. A copy of the written consent is available for review by the Editor-in-Chief of this journal.

## Competing interests

The authors declare that they have no competing interests.

## Authors' contributions

OEHS conceived of the case report and coordinated the write-up, HKS did the literature search and writing of the manuscript, SHS performed the surgical procedures and revised the manuscript, SOM carried out histopathological analyses and review of the manuscript, SZI revised the manuscript critically for important intellectual content. All authors read and approved the final manuscript.
